# Association between clinical outcome and microbiological findings in peritonsillar abscess - an observational study

**DOI:** 10.1007/s10096-025-05156-y

**Published:** 2025-05-14

**Authors:** Elin Lindberg, Ann Hermansson, David Nygren, Marie Gisselsson-Solén

**Affiliations:** 1 Department of Otorhinolaryngology, Head and Neck Surgery, Lund University, Lund, Sweden; 2 Division of Infection Medicine, Lund University, Lund, Sweden; 3 Department of Infectious Diseases, Skåne University Hospital, Malmö, Sweden

**Keywords:** *Fusobacterium necrophorum*, *Streptococcus pyogenes*, Peritonsillar abscess, Microbiology, Complications

## Abstract

**Background:**

Previous studies on causative pathogens in peritonsillar abscesses have yielded varying results. However, Group A streptococci (GAS) and *Fusobacterium necrophorum* have been identified frequently. The aim of this study was to investigate pathogens in peritonsillar abscesses in patients tested for both ß-hemolytic streptococci and *F. necrophorum*, and to investigate associations between pathogens and clinical outcome.

**Method:**

This retrospective observational study included all patients in the Skåne Region, Sweden (population 1.4 million) with a diagnosis of peritonsillar abscess between 2016 and 20, and in whom tests were performed for both ß-hemolytic streptococci (culture) and *F. necrophorum* (PCR). Exclusion criteria included previous (30 days) purulent complication to pharyngotonsillitis or antibiotic therapy. Chart review from 30 days prior to 6 months after the index visit was performed. Logistic regression was performed to evaluate the association between pathogens and complications, with negative microbiological findings set as reference category. Complications were defined as a composite outcome (0/1) of recurrent pharyngotonsillitis/peritonsillar abscess, other pharyngeal abscess or septic complications within 30 days (early), and 1–6 months (late).

**Results:**

In a total of 637 patients, *F. necrophorum* was identified in 210 (33%), GAS in 159 (28%) and GCS/GGS in 40 (6%) patients. *F. necrophorum* was most common in adolescents and young adults. Only *F. necrophorum* was associated with the development of either of early (OR 3.8 (2.0-7.1 95%CI)) and late complications (OR 2.5 (95%CI 1.3–4.9).

**Conclusion:**

*F. necrophorum* was the most commonly identified pathogen in peritonsillar abscesses. It was also the only pathogen associated with the development of complications.

**Supplementary Information:**

The online version contains supplementary material available at 10.1007/s10096-025-05156-y.

## Introduction

The most common complication of pharyngotonsillitis is a peritonsillar abscess (PTA) [1]. A peritonsillar abscess is a collection of pus between the palatine tonsil and its capsule [2]. Most patients who present with a PTA have a history of either recent PTA or pharyngotonsillitis, the latter usually within the preceding week [2–5]. However, Sanmark et al. [6] found that 14% of patients with PTA exhibited no tonsillar findings such as tonsillar erythema or exudate, suggesting that PTA could evolve de novo without previous pharyngotonsillitis.

People of any age and gender can develop a PTA. It affects men and women equally. As children, adolescents and young adults are most affected by pharyngotonsillitis, it is not surprising that most PTA patients are young, with a median age ranging from 16 to 36 years in previous studies [6–9]. 

Peritonsillar abscesses can be caused by numerous pathogens, including *Streptococcus pyogenes/*Group A streptococci (GAS) as well as Group C/G streptococci (GCS/GGS) [10]. Another historically less discussed pathogen is *Fusobacterium necrophorum*. Recent studies have shown *F. necrophorum* to be an important pathogen in both pharyngotonsillitis and PTA [7, 11, 12]. The microbial findings in PTAs have varied between studies. The prevalence rates of pathogens have ranged from 17 to 28% for GAS, 3–23% for *F. necrophorum* and 2–5% for GCS/GGS [5–8, 10, 13]. Recent studies have shown *F. necrophorum* to be the most frequent pathogen in PTAs and a main cause of pharyngotonsillitis among adolescents and young adults (15–30 years) [7, 14]. One study found that *F. necrophorum* was the causative pathogen in 28% of pharyngotonsillitis cases investigated for both beta-haemolytic streptococci and *F. necrophorum* and was associated with the development of complications, including recurrence of pharyngotonsillitis and PTA [12]. 

The primary aims of this study were to study the prevalence of pathogens and their association with complications in patients with PTA in whom microbiological testing for both β-hemolytic streptococci and *F. necrophorum* was performed. The secondary aim was to investigate the association between seasonal variation and the various pathogens.

## Materials and methods

### Study design and setting

This was a retrospective, observational study investigating the microbiological findings in PTAs, the prognosis, and clinical features in patients presenting to the Departments of Otorhinolaryngology, Head and Neck Surgery or emergency rooms at the hospitals in the Skåne region, Sweden, with a population of 1.4 million (2020) between 2016 and 2020. The standard treatment of a PTA in Sweden is to perform a puncture of the peritonsillar region under local anesthesia to try to obtain pus, and, if this is successful, to make an incision in order to drain the abscess properly. If no pus is obtained from the puncture, the same procedure is repeated the next day until either pus is obtained and an incision is made, or the peritonsillar swelling has disappeared. Children undergo a hot tonsillectomy rather than puncture/incision under local anesthesia.

### Case definition and exclusion criteria

Patients were eligible for inclusion if they had a diagnosis of PTA (ICD-10 code J36 or J369) and simultaneous microbiological analysis for both *F. necrophorum* (PCR) and ß-hemolytic streptococci (culture). Cases were categorized according to presence of the following pathogens: *F. necrophorum*, GAS, GCS/GGS (aggregate group) or negative. Exclusion criteria were prior treatment (30 days) with clindamycin, metronidazole, or ß-lactam antibiotic therapy, or previous (30 days) PTA or other purulent complication, defined as non-peritonsillar pharyngeal abscess, acute rhinosinusitis, acute otitis media, chronic tonsillitis, sepsis, or septic complications. Patients erroneously coded as PTA were also excluded. ICD-10 codes used to define eligibility and exclusion criteria are listed in Supplementary Appendix 1.

### Microbiological diagnostics

All microbiological analyses were performed using routine laboratory procedures at the Department of Clinical Microbiology in Lund, using ESwab test containing Liquid Amies medium and a regular flocked swab (Copan). For throat cultures, 30 µL of the sample was placed on Columbia agar plates containing sheep blood in the top layer, where the substrate was prepared using Columbia II agar (BD2997596). The plates were anaerobically incubated in an Electrotek anaerobic workstation (Electrotek Scientific) for a minimum of 16 h. Typical β-hemolytic colonies were Lancefield classified as GAS, GCS, or GGS using a Streptex Latex Agglutination Test, according to the manufacturer’s instructions (Remel; Thermo Scientific R30950501). Further species identification of GCS/GGS (e.g., *Streptococcus dysgalactiae* supsp. *equisimilis* and *dysgalactiae*,* Streptococcus equi*, and *Streptococcus canis*) with matrix-assisted laser desorption/ionization–time of flight mass spectrometry in throat cultures was not routinely performed. PCR for *F. necrophorum* was conducted as described by Nygren et al. [15]. If a rapid antigen detection test (RADT) for GAS had been performed and was positive, this also fulfilled the microbiological case definition for GAS.

### Primary and secondary outcomes

The primary outcome was the distribution of pathogens in PTAs and their association with complications. As defined by Klug et al. [16], “complication” was a composite outcome of recurrent PTA, pharyngotonsillitis, non-peritonsillar pharyngeal abscess and other septic complications following PTA, including Lemierre’s syndrome. Complications occurring within 1 (early) and after 1–6 months (late) were investigated separately. The secondary outcome investigated seasonal variation by pathogen.

### Variables

Demographic data, microbiological results, previous medical history, symptoms and clinical presentation, laboratory tests, vital parameters, medical and surgical treatment, information about hospital admission, complications and follow-ups were extracted from the medical charts. Information about prescribed antibiotics and ICD-10 diagnoses in the preceding 30 days were extracted from drug and diagnosis registries.

The medical history, symptoms and clinical findings were all based on the index visit. Only laboratory tests (CRP, RADT) performed within one day of the index visit were noted. The medical charts were reviewed for complications occurring within six months of the index visit.

Symptoms such as fever (≥ 38.0 °C) or unilateral pain were noted. If a symptom was not mentioned, we defined it as not present. Information about complications [16] or new episodes of PTA or pharyngotonsillitis during six months after the index visit was also extracted from the medical files. In the descriptive statistics, cases with two identified pathogens were presented as separate cases in each pathogen group. In the regression analyses, cases with coinfection were defined as *F. necrophorum* if present and as GAS if other β-hemolytic streptococci were also present. Sensitivity analyses were performed to assess any potential bias of these definitions of co-infections, and to assess the impact of mono- versus polymicrobial infection.

### Statistical analysis

Statistical analyses were performed using Stata 16.1 (College Station, TX, USA). The number of patients during the study period determined the sample size. In the primary analyses, the prevalence of pathogens was highlighted using descriptive statistics with counts and percentages. Logistic regression analyses were performed with the occurrence of complications (composite outcome) as the dependent variable. Crude odds ratios (OR) were analysed for the association between microbiological findings and the presence of complications, using negative PCR/culture as the reference group. These associations were then adjusted for age, gender and presence of any comorbidity (no/yes) according to Charlson comorbidity index [17]. 

Age was treated as a categorical variable, dividing the patients into three groups; 0–14, 15–40, and > 40 years. To analyse seasonal variation, monthly case counts of PTAs and distribution of cases due to *F. necrophorum* and GAS were presented. Summer was defined as the period April-September and winter as October-March. In a logistic regression model, the association between season and pathogen was investigated.

Due to the observational design, there was missing or unavailable data for laboratory values, medical history, and clinical information, e.g., CRP, history of smoking and vital parameters. No imputation was performed, since these variables were not the essential focus of the paper. Ethical approval was sought and given by the *National Ethics Board* in Sweden (no. 2017/971).

## Results

**Fig. 1:**
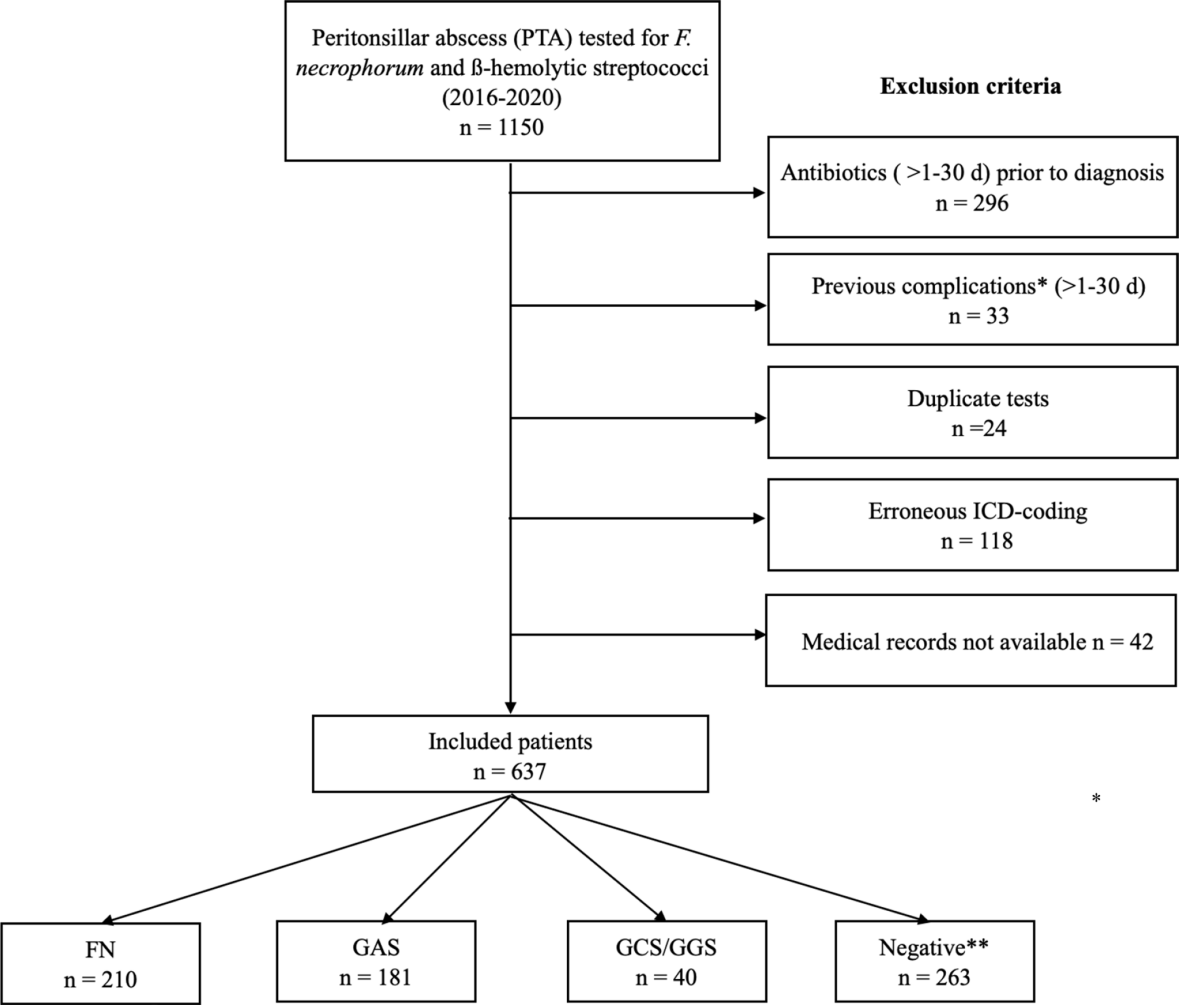
Flow chart of inclusion and exclusion criteria. Several patients had coinfections, causing the total to be > 637 cases. Previous complications* include peritonsillar abscess, non-peritonsillar pharyngeal abscess, acute rhinosinusitis, acute otitis media, chronic tonsillitis, sepsis, or septic complications. Abbreviations: *FN*,* Fusobacterium necrophorum*; GAS, group A streptococci; GCS, group C streptococci; GGS, Group G streptococci; ** Negative findings for the bacteria tested for, including ß-hemolytic streptococci and *F. necrophorum*

Initially, 1150 patients were identified and examined for eligibility. After applying exclusion criteria, 637 patients were included in the study (Fig. 1), corresponding to 13% of patients in the region of Skåne with a registered diagnosis code of J36/J369 during the study period (*n* = 5015).

### Baseline characteristics

Demographic data, symptoms, clinical findings, and management are presented in Table 1. Most patients were young adults, and there were no significant gender differences in general, or by pathogen. Patients with *F. necrophorum* and GCS/GGS were mainly adolescents and young adults, whereas patients with GAS and with negative findings were slightly older. Most of the patients were previously healthy, yet some had previous episodes of PTA, whereas only a few had undergone tonsillectomy. The symptoms and clinical findings listed in Table 1 were similar in all groups, except for temperature and CRP, which was lower in the group with negative PCR/culture. All patients received efficient antibiotic therapy and the number of surgical interventions was similar between groups (Table 1).

**Table 1:** Baseline characteristics and management of patients with peritonsillar abscess

	*Fusobacterium necrophorum* (*n* = 210)	GAS (*n* = 159)	GCS/GGS (*n* = 40)	Negative (*n* = 263)
Age, years (median, IQR)	22 (18–32)	36 (27–43)	25 (19–33)	40 (26–56)
Female, n (%)	113 (54%)	87 (55%)	24 (60%)	144 (55%)
Smoking, n (%)	15 (7%)	11 (7%)	3 (8%)	22 (8%)
Previous tonsillectomy, n (%)	8 (4%)	4 (3%)	2 (5%)	8 (3%)
Previous peritonsillar abscess, n (%)	37 (18%)	28 (18%)	9 (23%)	54 (21%)
Coinfection, GAS, n (%)	11 (5%)	-	0 (0%)	-
Coinfection, GCS/GGS, n (%)	24 (11%)	0 (0%)	-	-
Coinfection, *F. necrophorum*, n (%)	-	11 (7%)	24 (60%)	-
**Symptoms & findings**				
Dysphagia, n (%)	158 (75%)	114 (72%)	32 (80%)	174 (66%)
Unilateral pain, n (%)	130 (62%)	97 (61%)	26 (65%)	173 (66%)
Fever > 38, n (%)	110 (52%)	90 (57%)	22 (55%)	100 (38%)
Spontaneous rupture, n (%)	19 (9%)	14 (9%)	4 (10%)	26 (10%)
Days of symptoms (median, IQR)	4 (3–6)	5 (3–7)	3 (2–7)	4 (3–6)
Tonsillar bulging, n (%)	192/209 (91%)	145 (91%)	35 (88%)	231/262 (88%)
Trismus, n (%)	113 (54%)	84 (53%)	23 (58%)	136 (52%)
Lymphadenitis, n (%)	90/128 (70%)	75/97 (77%)	16/24 (67%)	97/152 (64%)
Uveal deviation, n (%)	52/209 (25%)	36 (23%)	9 (23%)	60 (23%)
CRP (median, IQR)*	117 (64–197)	101 (62–180)	174 (85–234)	76 (42–135)
**Management**				
Punction, n (%)	181 (86%)	132 (83%)	35 (88%)	221 (84%)
Incision, n (%)	76 (36%)	67 (42%)	9 (23%)	113 (43%)
Tonsillectomy à chaud, n (%)	7 (3%)	13 (8%)	2 (5%)	11 (4%)
Tonsillectomy within 6 months, n (%)	50 (24%)	22 (14%)	11 (28%)	35 (13%)
Hospital admission, n (%)	63 (30%)	46 (29%)	12 (30%)	59 (22%)

Abbreviations: IQR, interquartile range; GAS, group A streptococci; GCS, group C streptococci; GGS, Group G streptococci

Cases with coinfections are presented in multiple columns. If data is missing, it is presented with the denominator representing cases with complete data. * Available data from 433/637 (68%) patients

### Distribution of pathogens

*F. necrophorum* was the most commonly identified pathogen (210/637 (33%)), followed by GAS (181/637 (28%)) and GCS/GGS (40/637 (6%)) (Table 1). In 263/637 (41%), no pathogen was identified. In the cohort, 35/637 (5%) percent had polymicrobial findings. The most common coinfection was *F. necrophorum* and GCS/GGS, which was seen in a majority of cases where GCS/GGS were identified (24/40 (60%)).

### Complications

A complication (composite outcome) occurred within 30 days in 48/637 (8%) of patients. Patients infected with *F. necrophorum* had the highest complication rate during the first 30 days (39/210; 19%), whereas it was equally rare among patients with GAS (8/148; 5%), GCS/GGS (1/16; 6%) and patients with negative microbiological findings (15/263; 6%). Concerning late (1–6 months after the index visit) complications (composite outcome), these were again most common in patients with *F. necrophorum (*28/210; 13%) or GCS/GGS (2/16; 13%), followed by GAS (15/148; 10%) and negative microbiological findings (15/262; 6%).

The most common complications, during the first month as well as during the 1–6-month period, were recurrent PTA followed by recurrent tonsillitis (Table 2).

**Table 2:** Complication rate by pathogen following peritonsillar abscess

	*F. necrophorum* (*n* = 210)	GAS (*n* = 148)	GCS/GGS (*n* = 16)	Negative (*n* = 263)
**Complications** ^**a**^ **within 30 days of index visit**	39 (19%)	8 (5%)	1 (6%)	15 (6%)
Recurrence of pharyngotonsillitis, n (%)	9 (4%)	1 (1%)	1 (6%)	10 (4%)
Recurrence of peritonsillar abscess, n (%)	21 (10%)	4 (3%)	0%	6 (2%)
Other pharyngeal abscess, n (%)	9 (4%)	3 (2%)	0%	6 (2%)
Other septic complications, n (%)	2 (1%)	0%	0%	2 (1%)
**Late complications** ^**a**^ **(1–6 months) following index visit**	28 (13%)	15 (10%)	2 (13%)	15 (6%)
Recurrence of pharyngotonsillitis, n (%)	14 (7%)	10 (7%)	1 (6%)	6 (2%)
Recurrence of peritonsillar abscess, n (%)	16 (8%)	5 (3%)	2 (13%)	10 (4%)
Other pharyngeal abscess, n (%)	1 (0.5%)	0%	0%	0%
Other septic complications, n (%)	0%	0%	0%	0%

^a^ Complications were defined as a composite outcome of any recurrence of pharyngotonsillitis, peritonsillar abscess other abscess, or another septic complication following PTA, including Lemierre’s syndrome [16]

Abbreviations: GAS, group A streptococci; GCS, group C streptococci; GGS, Group G streptococci

Cases with coinfection were defined as *F. necrophorum* if present and as GAS if other β-hemolytic streptococci were also present

The development of complications within 30 days as well as the 1–6-month period following the index visit, was only associated with *F. necrophorum.* This association was not affected by adjusting for age, gender, and presence of any comorbidity (Table 3). The sensitivity analysis which investigated the impact of coinfections on associations with complications did not identify a notable impact on the associations presented above (Supplementary Appendix 2, Table 1).

**Table 3:** Associations between complications^a^ (within 30 d) and bacterial findings following peritonsillar abscess

	Crude OR (95% CI) *p*-value	Adjusted OR (95% CI) *p*-value	Complication^a^ rate (30 d), *n* (%)
**Bacterial findings**			
Negative (*n* = 263)	Reference	Reference	15/263 (6%)
*F. necrophorum* (*n* = 210)	**3.8 (2.0-7.1)** ***p*** **< 0.001**	**3.8 (1.9–7.4)** ***p*** **< 0.001**	39/210 (19%)
GAS (*n* = 148)	0.9 (0.4–2.3) *p* = 0.90	0.9 (0.4–2.3) *p* = 0.91	8/148 (5%)
GCS/GGS (*n* = 16)	1.1 (0.1–8.9) *p* = 0.93	1.1 (0.1–9.4) *p* = 0.90	1/16 (6%)
**Independent variables**			
Age category (< 15 years)	-	Reference	-
Age 15–40 years	-	1.3 (0.3–5.9) *p* = 0.71	-
Age > 40 years	-	1.0 (0.2-5.0) *p* = 0.97	-
Gender (female) (0/1)	-	0.9 (0.5–1.5) *p* = 0.60	-
Any comorbidity of the Charlson comorbidity index (0/1). (17)	-	1.9 (0.8–4.8) *p* = 0.17	-

^a^ Complications were defined as a composite outcome of any recurrence of pharyngotonsillitis, peritonsillar abscess other abscess, or another septic complication following PTA, including Lemierre’s syndrome, within 30 days [16]

Abbreviations: GAS, group A streptococci; GCS, group C streptococci; GGS, Group G streptococci; OR, odds ratio; CI, confidence interval

Cases with coinfection were defined as *F. necrophorum* if present and as GAS if other β-hemolytic streptococci were also present. Statistically significant associations, defined as *p* < 0.05, are highlighted in bold

**Table 4:** Associations between late complications (1–6 months) and bacterial findings following peritonsillar abscess

	Crude OR (95% CI) *p*-value	Adjusted OR (95% CI) *p*-value	Complication^a^ rate (30 d), *n* (%)
**Bacterial findings**			
Negative (*n* = 263)	Reference	Reference	15/263 (6%)
*F. necrophorum* (*n* = 210)	**2.5 (1.3–4.9)** ***p*** **= 0.005**	**2.1 (1.1–4.2)** ***p*** **= 0.03**	28/210 (13%)
GAS (*n* = 148)	1.9 (0.9–3.9) *p* = 0.10	1.7 (0.8–3.7) *p* = 0.16	15/148 (10%)
GCS/GGS (*n* = 16)	2.4 (0.5–11) *p* = 0.28	2.3 (0.5–11) *p* = 0.31	2/16 (13%)
**Independent variables**			
Age category (< 15 years)	-	Reference	-
Age 15–40 years	-	1.6 (0.4-7.0) *p* = 0.54	-
Age > 40 years	-	0.8 (0.2–3.7) *p* = 0.74	-
Gender (female) (0/1)	-	0.9 (0.5–1.6) *p* = 0.76	-
Any comorbidity of the Charlson comorbidity index (0/1) (17)	-	1.7 (0.7–4.5) *p* = 0.27	-

^a^ Late complications were defined as a composite outcome of any recurrence of pharyngotonsillitis, peritonsillar abscess other abscess, or another septic complication following PTA, including Lemierre’s syndrome [16], 1–6 months following the index visit

Abbreviations: GAS, group A streptococci; GCS, group C streptococci; GGS, Group G streptococci; OR, odds ratio; CI, confidence interval

Cases with coinfection were defined as *F. necrophorum* if present and as GAS if other β-hemolytic streptococci were also present. Statistically significant associations, defined as *p* < 0.05, were highlighted in bold

Abbreviations: GAS, group A streptococci; GCS, group C streptococci; GGS, Group G streptococci

### Seasonal variation

**Fig. 2:**
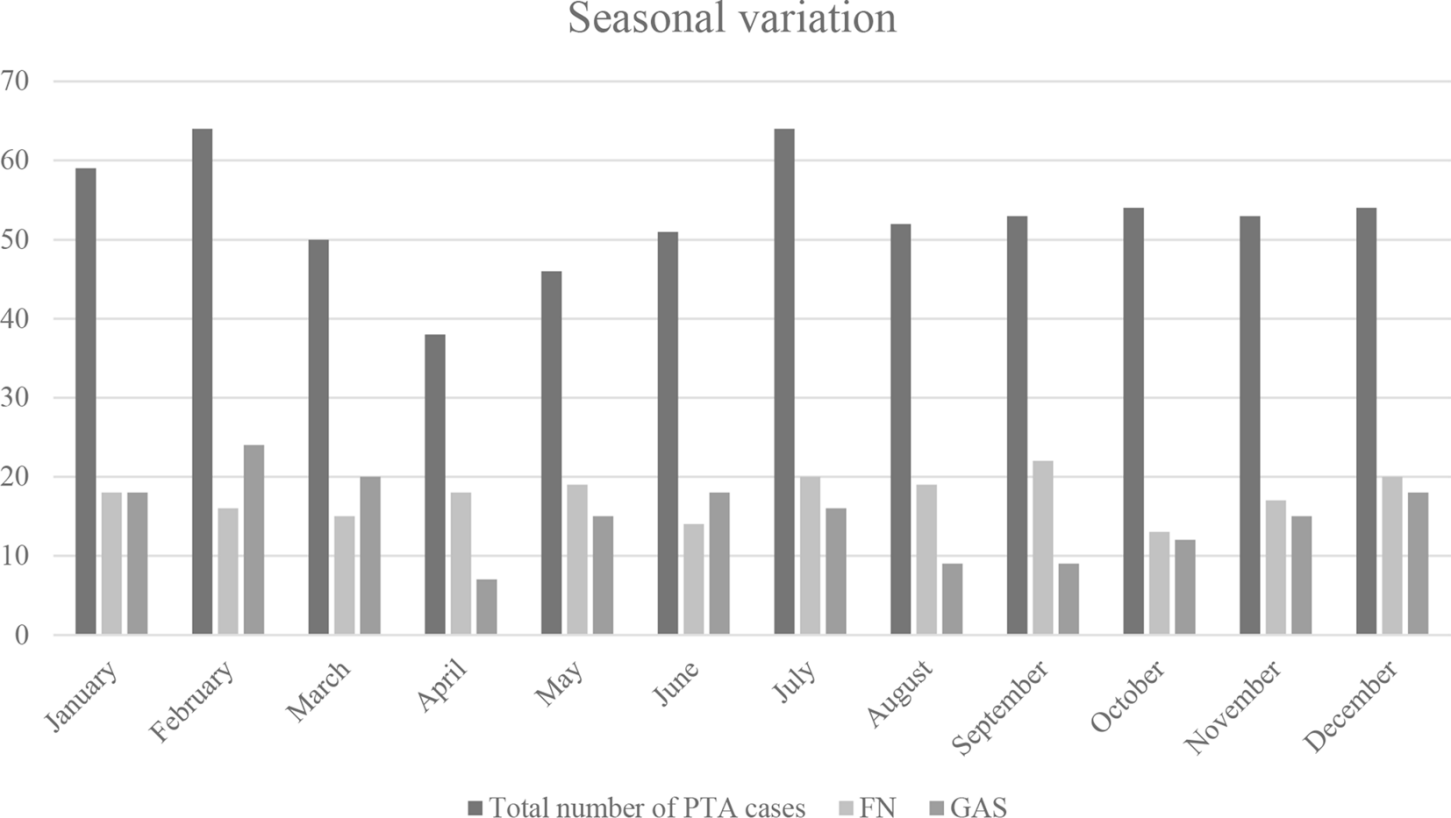
Monthly distribution of patients with peritonsillar abscess. Abbreviations: FN, *Fusobacterium necrophorum*; GAS, Group A streptococci; PTA, peritonsillar abscess

Findings concerning seasonal variation is presented in Fig. 2. GAS was less prevalent in the summer, whereas the opposite was true for *F. necrophorum*. The odds for GAS as a cause of PTA was higher in winter (OR 1.5 (1.03–2.1), *p* = 0.032). There was weak evidence that *F. necrophorum* was more prevalent during the summer (OR: 1.4 (0.99–1.9), *p* = 0.054).

## Discussion

This study, performed on a group of patients analysed for both GAS and *F. necrophorum*, aimed to describe the microbiology of PTAs and to investigate the association between pathogen and subsequent development of complications. In this group of patients, *F. necrophorum* was the most prevalent pathogen (33%), followed by GAS (28%) and GCS/GGS (6%). *F. necrophorum* was commonly found in adolescents and young adults and was the only pathogen found to be associated with the development of complications. GAS had a less distinct age distribution.

Previous studies have shown varying prevalence rates of *F. necrophorum* in PTA (3–23%) [5–7, 13, 18]. The distribution of pathogens in this study agrees well with a similar retrospective study from Denmark, where *F. necrophorum* was the most common pathogen (23%), followed by GAS (17%) and GCS/GCS (5%) [7]. Interestingly, the high prevalence of *F. necrophorum* in these two Scandinavian studies contrasts findings in other studies. In a Finnish study a prevalence rate of *F. necrophorum* of 7% was seen, however the study size was small (*n* = 58) [6]. Studies from Taiwan and Poland have yielded prevalence rates of *F. necrophorum* of 3–4% [5, 18]. Thus, the findings in our retrospective study have not been repeated outside Scandinavia. Potentially, *F. necrophorum* have greater geographical variation than GAS. While this has been suggested for tonsillar carriage, geographical differences remain insufficiently studied for throat infections [15]. The use of culture or PCR for detection of *F. necrophorum* might also impact detection levels, yet previous data are inconsistent [19, 20]. It is important to notice that the present study constitute only a subset (13%) of all patients with PTA as it only included patients in whom microbiological investigation for both *F. necrophorum* and beta-haemolytic streptococci had been performed. Consequently, our inclusion criteria has an inherent risk of selection bias, since suspicion of *F. necrophorum* might have existed. Our results regarding the age distribution correlate well with previous findings, showing a clear age distribution in *F. necrophorum* infections, with a higher prevalence among adolescents and young adults [12, 21].

The prevalence of GAS in the present study (28%) was in concordance with previous studies (17–28%) [5–8, 10, 13]. It is possible that the study design underestimated GAS prevalence in PTA, as patients with a positive RADT for GAS would typically not require further microbiological investigation, and would thus not be included in our study. The prevalence of GCS/GGS (6%) in this study was also similar to previous studies (2–5%) [6, 8, 13, 18]. Co-infections were generally rare, with the exception of GCS/GGS, where a majority of findings were in patients also positive for *F. necrophorum*.

In addition to describing the distribution of pathogens in PTA, we aimed to investigate their association with the clinical outcome during the first six months after the infection. Importantly, no differences were seen between patients in terms of medical or surgical treatment upon presentation, except for slightly lower rates of incisions performed among patients with GCS/GGS. Complication rates overall were low, yet interestingly, only *F. necrophorum* was found to be associated with the development of complications within 30 days as well as for late complications (1–6 months). The composite outcome included recurrence of pharyngotonsillitis and PTA, which were found to be the most common complications, as well as 17 distinct types of complications following PTA, including other pharyngeal abscesses, Lemierre’s syndrome, necrotizing fasciitis and mediastinitis, as defined by Klug et al. [16]. Klug et al. were unable to quantify the prevalence of complications to PTA, but stated that it was very low, which correlates well with our findings. Analyses were adjusted for age, in addition to sex and comorbidities. The age categorization was designed to account for that most cases of *F. necrophorum* and PTA occur between ages of 15–40 years [7, 22], but also for potential differences in management between younger and older patients, which could impact clinical outcomes. However, no associations were found between age group and outcomes. While not defined as an outcome, a pattern was seen where patients with *F. necrophorum* or GCS/GGS more commonly underwent a late tonsillectomy. Among patients with GCS/GGS who needed a tonsillectomy within six months, 6/11 (55%) had a co-infection on presentation with *F. necrophorum*. This overrepresentation of late surgical procedures among patients with *F. necrophorum*, were interpreted as being performed due to recurrences.

Importantly, our study design only identified hospital visits/admissions, and not visits to primary care. Therefore, our data on the occurrence of recurrent pharyngtonsillitis is probably an underestimation. Finally, it could be argued that microbiological testing in patients with PTA is unnecessary, since it does not affect the management of the patient, and, indeed, in clinical practice, microbiological analysis is only performed in a minority of patients. Nevertheless, from this study, it is obvious that the presence of *F. necrophorum* is in fact a predictor of higher recurrence rates in the short term as well as in the long term.

The incidence of PTAs was stable over the year, when comparing winter with summer months, a finding that correlates well with previous studies [18, 23]. Yet, similar to previous research [24], GAS was found to be a more common cause during the winter months, whereas there was a tendency for *F. necrophorum* to be found more frequently during the summer.

The most important limitation of this study is that the population represented a select group of PTA patients, i.e., those who were tested for both beta-haemolytic streptococci and *F. necrophorum*, which limits generalizability. Most patients with PTA do not undergo any microbiological testing at all. Consequently, prevalence rates of pathogens presented here might be overestimations. Also, when investigating the association between pathogen and complications, we defined any positive finding of *F. necrophorum* as *F. necrophorum*, regardless of any simultaneous presence of GAS or GCS/GGS. This could have biased the interpretation of our findings, should coinfections be more strongly associated with complications. Importantly, our sensitivity analyses negated a notable impact of coinfections. In addition, the retrospective design limited the availability of background data, including previous medical history and clinical presentation. Given that we defined symptoms not mentioned in medical records as not present, it is possible that they were underestimated. In addition, we could not systematically investigate, for instance, smoking or dental disease as factors associated with recurrence of PTA.

Interestingly, our study echoes the findings of the retrospective study on causes of peritonsillar abscess in Denmark, suggesting *F. necrophorum* as a main cause of PTA in Scandinavia [7]. Future prospective studies on PTA are needed to determine the true prevalence of pathogens causing PTA. The association between *F. necrophorum* and complications, short and long term, also highlights the significance of *F. necrophorum* beyond being a primary causative agent. While testing for *F. necrophorum* in PTAs might identify patients at higher risk for complications, any clinical consequence of the test results beyond informing patients of the increased risk is not evident today. No study has yet investigated the efficacy of antibiotics in *F. necrophorum* throat infections. However, resistance to penicillin in invasive isolates of *F. necrophorum* is rarely seen in Sweden (1%) and penicillin is likely to be effective also in vivo [22]. Nevertheless, future studies are needed to evaluate the best antibiotic therapy of *F. necrophorum* infections. In PTAs caused by *F. necrophorum*, the efficacy of penicillin should be compared to either combination therapy with metronidazole or to monotherapy with clindamycin, preferably in a randomized controlled trial, where optimal duration of therapy should be investigated as well. Such a study would need to include culture of *F. necrophorum* in addition to PCR to assess antimicrobial susceptibility and to compare sensitivity of diagnostic methods.

In conclusion, in this observational, retrospective study of microbiologically investigated patients with PTA, *F. necrophorum* was the most prevalent pathogen followed by GAS and GCS/GGS. *F. necrophorum* affected adolescents and young adults without any gender difference. Complications were rare and most often occurred in the form of a recurrent pharyngotonsillitis or PTA. The only pathogen found to be associated with complications was *F. necrophorum*. These findings further establish *F. necrophorum* as a main pathogen in PTA beside GAS. Future studies are needed to assess the generalizability of these findings outside of Scandinavia.

## Electronic supplementary material

Below is the link to the electronic supplementary material.

Supplementary Material 1

## Data Availability

No datasets were generated or analysed during the current study.
