# Supplementary material for: Association between clinical outcome and microbiological findings in peritonsillar abscess - an observational study
Source: Eur J Clin Microbiol Infect Dis. 2025 May 14;44(8):1925–33. doi: 10.1007/s10096-025-05156-y (PMC12321682; doi:10.1007/s10096-025-05156-y)
Supplement: Supplementary file 1 — Supplementary Material 1 [file 10096_2025_5156_MOESM1_ESM.docx]

**Sensitivity analysis – comparing mono- and coinfections**

**Supplementary table 1:1**

| **Supplementary table 1:1** | Crude OR^a^ (95% CI)  p-value | Adjusted OR^a^  (95% CI)  p-value |
| --- | --- | --- |
| **Bacterial findings** |  |  |
| Negative (n=263) | Reference | Reference |
| *F. necrophorum* monoinfection (n=175) | **3.6 (1.9-6.8)**  **p<0.001** | **3.6 (1.8-7.3) p<0.001** |
| GAS monoinfection (n=148) | 0.9 (0.4-2.3) p=0.90 | 1.0 (0.4-2.3)  p=0.92 |
| GCS/GGS monoinfection (n=16) | 1.1 (0.1-8.9) p=0.93 | 1.1 (0.1-9.3)  p=0.90 |
| *F. necrophorum* & GAS (n=11) | 1.7 (0.2-14) p=0.64 | 1.7 (0.2-15)  p=0.1 |
| *F. necrophorum* & GCS/GGS (n=24) | **6.8 (2.4-19)**  **p<0.001** | **6.6 (2.2-19)**  **p=0.001** |
|  |  |  |
| **Independent variables** |  |  |
| Age category (<15 years) | - | Reference |
| Age 15-40 years | - | 1.3 (0.3-6.1)  p=0.70 |
| Age >40 years | - | 1.1 (0.2-5.5)  p=0.90 |
| Gender (female) (0/1) | - | 0.9 (0.5-1.5)  p=0.65 |
| Any comorbidity of the Charlson comorbidity index (0/1) (17) | - | 1.8 (0.7-4.5)  p=0.20 |

**Supplementary table 1:1.** Associations between complications^a^ (within 30 d) and bacterial findings following peritonsillar abscess

^a^ Complications were defined as a composite outcome of any recurrence of pharyngotonsillitis, peritonsillar abscess other abscess, or another septic complication following PTA, including Lemierre’s syndrome, (16) 1-6 months following the index visit.

within 30 days.

Abbreviations: GAS, group A streptococci; GCS, group C streptococci; GGS, Group G streptococci; OR, odds ratio; CI, confidence interval

Cases were defined as monoinfection with *F. necrophorum*, GAS or GCS/GGS or co-infection with *F. necrophorum* & GAS or GCS/GGS. There were no coinfections with GAS and GCS/GGS. Statistically significant associations, defined as p<0.05, were highlighted in bold.

| **Supplementary table 1:2** | Crude OR^a^ (95% CI)  p-value | Adjusted OR^a^ (95% CI)  p-value |
| --- | --- | --- |
| **Bacterial findings** |  |  |
| Negative (n=263) | Reference | Reference |
| *F. necrophorum* monoinfection (n=175) | **2.5 (1.3-4.9)**  **p=0.008** | **2.1 (1.02-4.3)**  **p=0.045** |
| GAS monoinfection (n=148) | 1.9 (0.9-3.9)  p=0.10 | 1.7 (0.8-3.7)  p=0.16 |
| GCS/GGS monoinfection (n=16) | 2.4 (0.5-11)  p=0.28 | 2.3 (0.5-11.1)  p=0.31 |
| *F. necrophorum* & GAS (n=11) | 3.7 (0.7-19)  p=0.12 | 3.4 (0.7-17.9)  p=0.14 |
| *F. necrophorum* & GCS/GGS (n=24) | 2.4 (0.6-8.8)  p=0.20 | 1.8 (0.5-6.8)  p=0.40 |
|  |  |  |
| **Independent variables** |  |  |
| Age category (<15 years) | - | Reference |
| Age 15-40 years | - | 1.6 (0.4-7.0)  p=0.54 |
| Age >40 years | - | 0.7 (0.2-3.7)  p=0.72 |
| Gender (female) (0/1) | - | 0.9 (0.5-1.6)  p=0.70 |
| Any comorbidity of the Charlson comorbidity index (0/1) (17) | - | 1.7 (0.7-4.5)  p=0.27 |

**Supplementary table 1:2.** Associations between late complications (1-6 months) and bacterial findings following peritonsillar abscess

^a^ Late complications were defined as a composite outcome of any recurrence of pharyngotonsillitis, peritonsillar abscess other abscess, or another septic complication following PTA, including Lemierre’s syndrome, (16) 1-6 months following the index visit.

Abbreviations: GAS, group A streptococci; GCS, group C streptococci; GGS, Group G streptococci; OR, odds ratio; CI, confidence interval

Cases were defined as monoinfection with *F. necrophorum*, GAS or GCS/GGS or co-infection with *F. necrophorum* & GAS or GCS/GGS. There were no coinfections with GAS and GCS/GGS . Statistically significant associations, defined as p<0.05, were highlighted in bold.
